# Impairment of maturation of BMP-6 (35 kDa) correlates with delayed fracture healing in experimental diabetes

**DOI:** 10.1186/s13018-020-01705-7

**Published:** 2020-05-24

**Authors:** Qidong Guo, Weijie Wang, Rami Abboud, Zheng Guo

**Affiliations:** 1 Department of Orthopaedics, Second Hospital of Shanxi Medical University, 382 Wuyi Road, Taiyuan, 030001 Shanxi China; 2 Department of Orthopaedic and Trauma Surgery, Medical School, University of Dundee, Dundee, UK; 3 Department of Anaesthesia, Second Hospital of Shanxi Medical University, 382 Wuyi Road, Taiyuan, 030001 Shanxi China

**Keywords:** BMP-6, Delayed union, Diabetes, Fracture, Healing, Nonunion

## Abstract

**Background:**

Although it is known that diabetes interferes with fracture healing, the mechanisms remain poorly understood. The aim of this study was to investigate the correlation of BMP-6 and BMP-9 with the impairment in fracture healing in diabetes, by analyses of the difference in size and calcification of the callus, mechanical endurance, and expressing BMP-6 and BMP-9 in the callus, using a clinical related diabetic rodent model.

**Methods:**

We evaluated femur fracture healing by quantification of size and calcification of the callus by X-ray, histological and histochemical images, loading capacity of the fractured bone, and amount of BMP-6 in the callus and the bones using Western blot assay.

**Results:**

Significant upregulation of BMP-6 in the callus and the fractured bones of both non-diabetic and the diabetic animals was observed, at the end of the second and the fourth weeks after fracture. However, significantly lower levels of BMP-6 at 35 kDa with smaller sizes of calcified callus and poor loading capacity of the healing bones were detected in the diabetic animals, compared to the non-diabetic controls. The impairment of the maturation procedure of BMP-6 (35 kDa) from precursors may be underlying the downregulation of the BMP-6 in diabetic animals.

**Conclusions:**

It could be concluded that the delayed fracture healing in the diabetic animals is correlated with deficiency of BMP-6 (35 kDa), which may be caused by impairment of maturation procedure of BMP-6 from precursors to functioning format. This is a primary study but an important step to explore the molecular pathogenesis of impairment of fracture healing in diabetes and to molecular therapeutic approach for the impairment of fracture healing.

## Background

Diabetes is a major challenger for human health. More than 400 million people are living with the disease and it is estimated that the number will rise to 700 million by 2045 [1]. Cumulating evidence indicates that diabetes impairs bone health and fracture healing [2]. Among the diabetes-related comorbidity, low energy trauma fracture is one of the major causes of morbidity and mortality of diabetic patients, due to the increased fracture risk and the impaired healing procedure, in comparison with non-diabetic cohort [3]. Clinical studies indicate a significantly higher incidence of delayed union and nonunion after fracture in diabetic patients, compared to the nondiabetic ones [4, 5]. The fracture nonunion, rating at 5–10% [6], is still a major complication causing longer physical disability, pain, mental dysfunction, and expenditure on the treatment world widely. The mechanism underlying the nonunion was thought to be mainly associated with severity of injury in the fracture site and the mode of surgical treatment [7, 8]. However, progression to nonunion is not fully explained by these factors alone [5]. Although a considerable effort has been made to identify the impact of diabetes on osteoblasts and bone formation [9–11], there have been few studies that investigate the molecular mechanism by which diabetes affects the process of fracture healing [12, 13]. Therefore, exploring the molecular, pathogenetic changes in the healing bone in diabetes is important for further understanding of the pathology of impairment of the fracture union.

Bone morphogenetic proteins (BMPs), as a member of the transforming growth factor-β (TGF-β) superfamily, mediate multiple biological processes including regulation of bone formation [14]. BMP-2 has been locally applied to achieve bone regeneration in diabetes [15]. However, the effect of other BMPs-6 and BMP-9 on fracture healing in diabetes has not been reported. In this study, we investigated the difference in expressions of BMP-6 and BMP-9 in the healing femurs between diabetic and non-diabetic animal models, while the change in radiological, histological, and mechanical parameter was evaluated, making an attempt to reveal molecular pathological impact of BMP-6 and BMP-9 on the fracture healing.

## Method and material

The study was conformed to the Guide for the Care and Use of Laboratory animals (Copyright 2011 by the National Academy of Sciences) and approved by the Academy and Ethics Committee of University of Dundee.

### Experimental protocol

Healthy male Sprague-Dawley rats weighing 255 ± 7 g were randomly assigned to diabetic group (*n* = 22) and age-matched non-diabetic control group (*n* = 22), according to previous study [13] and statistical power analysis. Diabetes was induced by intraperitoneal injection of streptozotocin (STZ). Femur fracture was induced 2 weeks after induction of diabetes. The difference in the healing of femur fracture between diabetic and non-diabetic rats was evaluated by X-ray examination at the end of the second, the fourth, and the eighth weeks after induction of the fracture. The mechanical test was performed at the end of the second and the fourth weeks after fracture. The expressions of BMP-6 and BMP-9 in the healing callus and the bones were quantitatively analyzed in both of diabetic and non-diabetic animals.

### Diabetes model

**Fig. 1:**
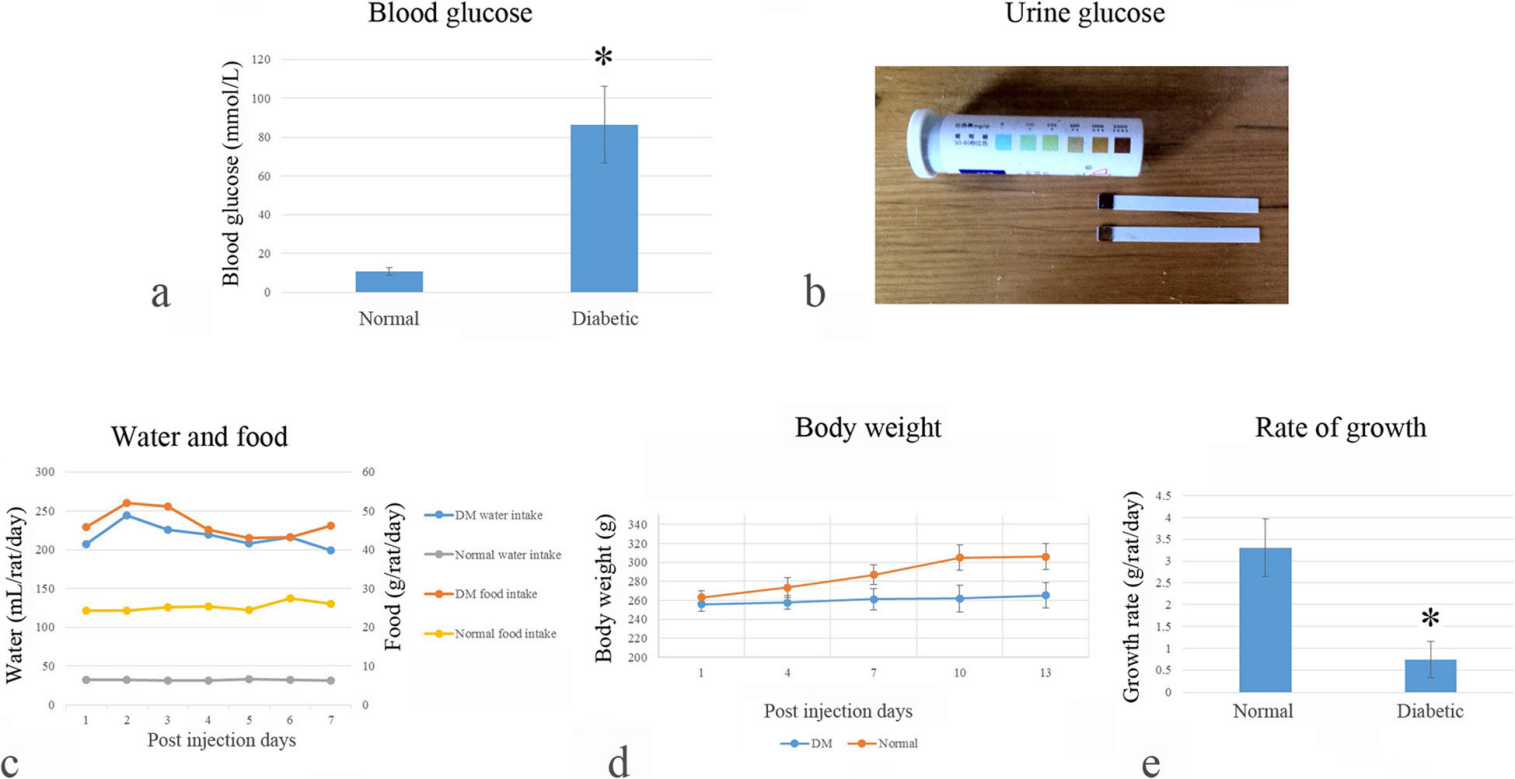
The diabetic model building. Rats showed typical diabetic symptoms including high fasting blood glucose (**a**), urine glucose (**b**), polyphagia, polydipsia (**c**), loss of body weight (**d**), and lower growth rate (**e**)

The animals assigned to diabetic group were treated with intraperitoneal injection of STZ (50 mg/kg, dissolved in 0.1 mol/L citrate buffer at pH 4.5) after 16-h fasting. Drinking water was supplied immediately after the treatment, while food was supplied 3 h after the injection of STZ. Diabetes was defined by a sustained blood glucose concentration greater than 16.7 mmol/L [16] 24 h after the injection of STZ, while those that showed blood glucose less than 16.7 mmol/L were excluded from further experiment. Body weight, food, and water consumption of the animals were closely monitored. Glucoses in the blood and urine were tested on the first post-injection day and then once every 3 days (Fig. 1).

### Fracture model

At the end of the second week after the induction of diabetes, transverse femoral fracture was induced. The animals were scheduled on 16-h fasting before fracture. The anesthesia of the animal was induced by inhalation of 5% of sevoflurane (delivered in 100% of oxygen) and maintained with 3.5~5% of sevoflurane, while spontaneous respiration of the animal was maintained. Gentamicin (8000 IU, 0.2 mL) was used (injected subcutaneously) to prevent infection. Left sciatic nerve was blocked with bupivacaine (0.125%, 1 mL). The left thigh of the animal was cleaned, shaved, and sterilized with iodophor. A 10 mm incision was made medial to the ligamentum patellae and then the tendon was bluntly dissected with mosquito-type hemostatic forceps. The ligamentum patella was drawn laterally with double 1-0 suture to expose the joint capsule. Then the capsule was opened with mosquito-type hemostatic forceps to expose the patellofemoral groove. A K-Wire (40 mm) was drilled into the marrow cavity in a retrograde fashion from the groove, as an internal fixation. The drilling was stopped when met resistance, which meant the distal end of the pin had been drilled into proximal cortex of the femur. The incision was closed with 4-0 suture. Then the fixed thigh was placed in abduction and externally rotated position on the guillotine system, with great trochanter on one supporter and supracondyle on the other. The impact distance of the blade was set at one-half of the long diameter of the contralateral femur. Then a 500-g weight fell freely from 300 mm height to drive the blade down, a mid-diaphyseal fracture was induced. The animal was allowed to recover from the anesthesia and the surgery. The data were not included in this report for the animals that died at any time before the end of the experiment observation.

### Radiology

Seven animals in each of the diabetic and the non-diabetic groups were scheduled for X-ray examination, at the end of the second and the eighth weeks following fracture, while eight animals in each of the diabetic and non-diabetic groups received radiological evaluation, at the end of the fourth week. X-ray examinations (Philips® Medical system, Nederland) were carried out for the animals under anesthesia (with 25% urethane, 1.25 g/kg, i.p.), in a supine position with the fractured thigh in abduction and externally rotated position, at the end of the second, the fourth, and the eighth post-fracture weeks. The size and calcification ratio of callus were evaluated and analyzed with ImageJ® software (National Institutes of Health, USA).

### Mechanical test

The fractured femurs were collected from nine animals (non-diabetic, *n* = 5 and diabetic, *n* = 4), at the end of the second week after surgery and from 11 animals (non-diabetic, *n* = 5 and diabetic, *n* = 6) 4 weeks following fracture, after euthanasia of the animals. A three-point bending system was employed to evaluate the pressure taken to break the healing callus.

### Histological and immunohistochemistry assay

Two fractured femurs were collected after euthanasia of the animals from each experimental group. The samples were then fixed in 4% of paraformaldehyde solution for 72 h. The distal and proximal ends of the femurs were cut off then the mid-shaft calluses were decalcified in 10% ethylene diamine tetraacetic acid (EDTA) solution at a temperature of 40 °C for 1 week. Then the calluses were cut into 5-μm-thick sections using a cryostat (Leica CM 1850, Germany). The sections were assigned for hematoxylin-eosin (HE) stain and immunohistochemistry (IHC) assay.

For immunohistochemistry assay, the sections were processed with primary anti-BMP-6 and anti-BMP-9 (Santa Cruz Biotechnology Inc., California, USA) and the secondary antibodies (Zhongshan Biotechnology Inc., Beijing, China) according to the instructions of the suppliers. Chromagen 3,30-diaminobenzidine (Zhongshan Biotechnology Inc., Beijing, China), brown reaction product, was added as the final substrate to visualization of the antigens.

### Western blot

The fractured and the intact (the contralateral) femurs were collected from ten animals of non-diabetic (*n* = 5) and diabetic groups (*n* = 5), 2 weeks after surgery and from other 12 animals of non-diabetic (*n* = 6) and diabetic groups (*n* = 6), 4 weeks after induction of fracture. The mid-shaft callus and bones (in fractured femurs) or the bones in the corresponding position (in the intact femurs) were resected and grinded in liquid nitrogen (− 196.56 °C). Then the total protein was extracted from the callus for Western blot assay, as we previously reported [17]. The ECL chemical luminescence method was employed to detect the labeled antigen. The values of optical density of all blotting bands were measured with Quantity One® software (Bio Rad, USA). The values of BMP-6 and BMP-9 were standardized by being divided by the values of GAPDH. Then the standardized values were analyzed.

### Statistical analysis

The sample size of the experiments was determined using G*Power software (version 3.1.9.7). According to the primary observations in the pilot experiments, the standardized effect size was calculated based on the differences between the means of the experimental groups and the standard deviations. The significant level, ‘α err prob’ at 0.05 was chosen. Data of the results are presented as mean ± standard deviation (S.D.) and compared between two groups using the Student’s *t* test, or by one-way analysis of variance (ANOVA), using Tukeys’ multiple comparison test for post hoc analysis, with *p* < 0.05considered statistically significant.

## Results

### Diabetic model

Data reported here were collected from the 22 animals that presented significant high level of glucose in blood and in urine (Fig. 1a, b, *p* < 0.01) and lived through the observation period. The consumption of food and drinking water was significantly increased while the body weight and growth rate were significantly reduced in the diabetic animals, compared with the non-diabetic controls (Fig. 1c–e, all *p* < 0.01).

### Radiological evaluation

**Fig. 2:**
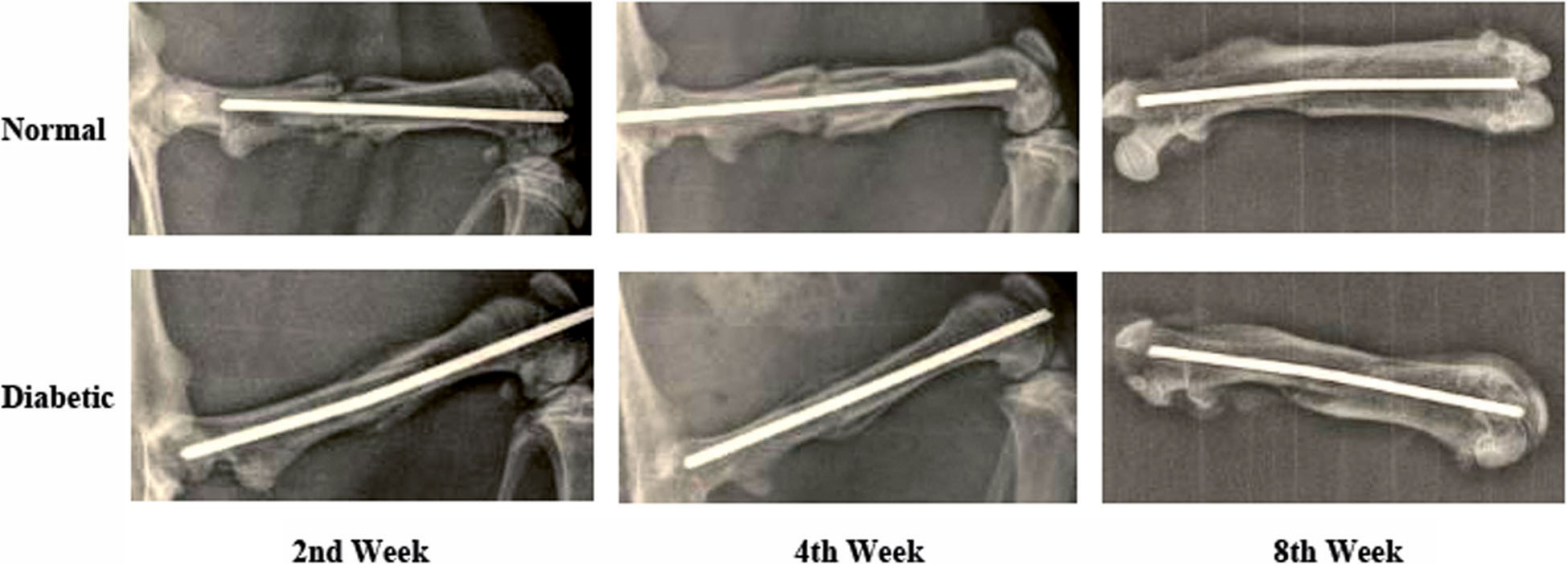
Radiological evaluations of the healing. Smaller calluses with few calcified calluses were observed in diabetic animals, compared with that in normal controls. At the eighth post-fracture week, the non-diabetic group had almost finished fracture remodeling while diabetic one had not

The results showed that, at the second and the fourth weeks after fracture, the calluses of the fractured femurs in the diabetic animals were much weaker than their non-diabetic counterparts, by presenting predominant fibrous calluses with few calcified calluses (Fig. 2). At the eighth post-fracture week, the non-diabetic group had almost finished fracture remodeling while diabetic one had not (Fig. 2). Thus, the results of radiological exams at the end of the fourth week were employed in comparative and quantitative analyses. The size of callus was presented as the ratio of the callus diameter to the femoral diameter. Based on the analysis, the significantly smaller size of calluses was observed in diabetic animals, compared to that of the non-diabetic group (non-diabetic vs diabetic, 2.14 ± 0.18 vs. 1.64 ± 0.14, *n* = 8, *p* < 0.05). The calcification ratio of diabetic calluses was also significantly lower than that of non-diabetic calluses (non-diabetic vs. diabetic, 60.61 ± 2.13% vs. 53.40 ± 6.49%, *n* = 8, *p* < 0.05).

### Mechanical evaluation

The mechanical endurance of diabetic callus was markedly reduced than that of non-diabetic callus at both the second and the fourth post-fracture weeks. But only the difference was found statistically significant, between the non-diabetic and diabetic groups at the end of the fourth week of fracture (non-diabetic vs. diabetic: 217.49 ± 39.63 kPa vs. 161.85 ± 38.33 kPa, *p* < 0.05, Table 1).

**Table 1:** Mechanical endurance of the fractured bones (kPa)

Groups	2nd week		4th week	
	Non-DM (*n* = 5)	DM (*n* = 4)	Non-DM (*n* = 5)	DM (*n* = 6)
	92.40	30.19	170.88	211.92
	110.50	28.72	260.58	212.80
	12.90	48.66	230.39	108.45
	61.55	87.93	255.30	148.61
	130.73		170.30	151.60
				137.73
Mean	81.62	48.88	217.49	161.85*
SD	41.20	23.88	39.63	38.33

*p < 0.05, compared with the ‘non-DM’ at the end of fourth week after fracture

### Histological and immunohistochemistry assay

**Fig. 3:**
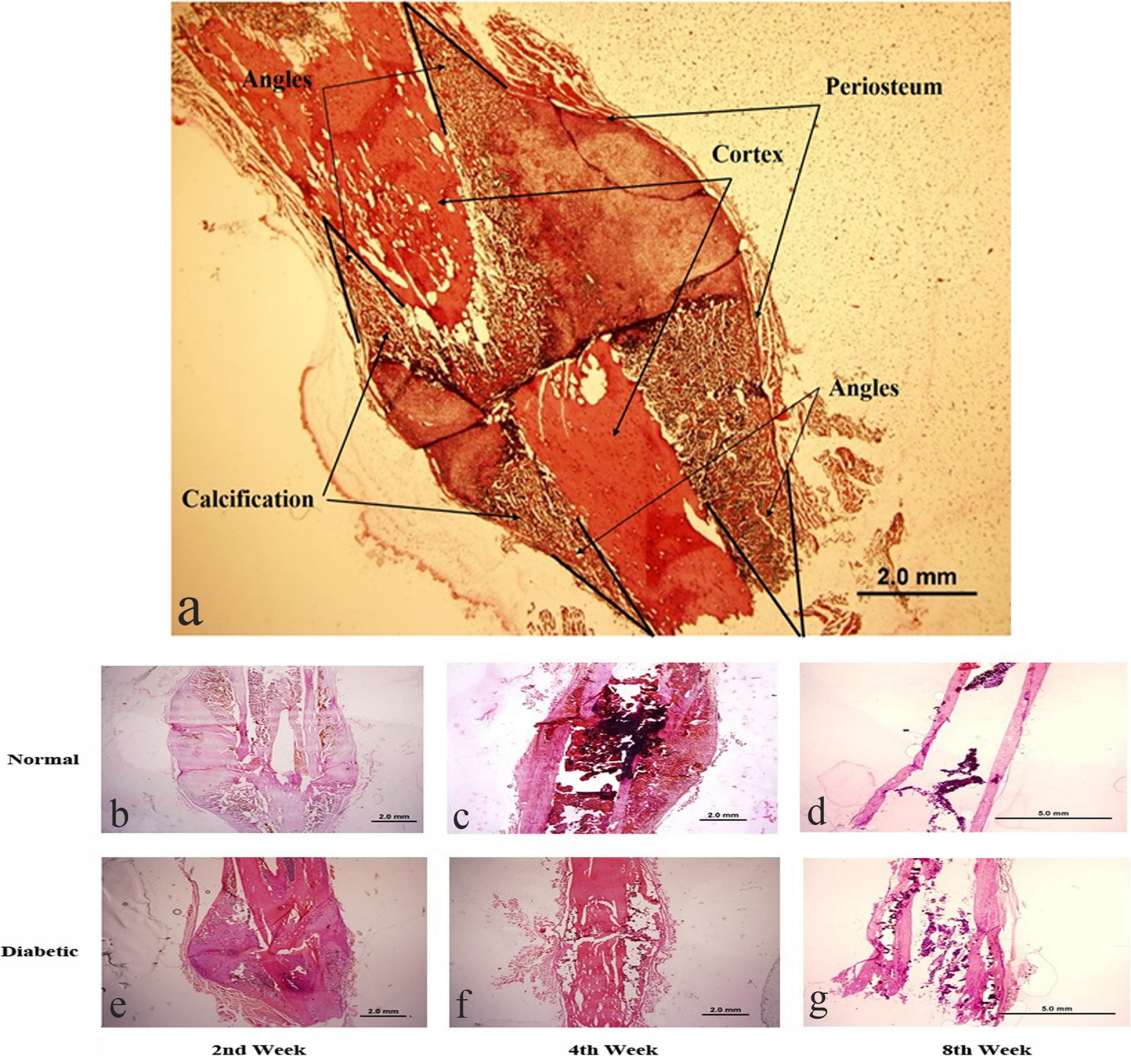
HE stained healing calluses. Fracture end surrounded by a spindle red-stained callus with calcification started from the four angles formed by periosteum and cortex (**a**). Smaller size of calluses was found in diabetic animals at the end of the second and fourth week after fracture (**e**, **f**) than that of the normal group (**b**, **c**). At the end of the eighth week, calluses from the normal group had finished reconstruction (**d**) while calluses from the diabetic group had not (**g**)

It can be clearly seen that the fracture ends were surrounded by a spindle red-stained callus. Calcification started from the four angles formed by periosteum and cortex (Fig. 3a). The (red-stained) callus was significantly smaller in the diabetic group at the second and the fourth post-fracture weeks (Fig. 3b and c vs. e and f). At the eighth week after the fracture, the fracture in the non-diabetic control was well healed and showed typical double-track sign as intact bones (Fig. 3d). However, the cortex of the fracture bones in diabetic animals was still under remodeling (Fig. 3g), which was consistent with the results of radiological evaluation (Fig. 2).

**Fig. 4:**
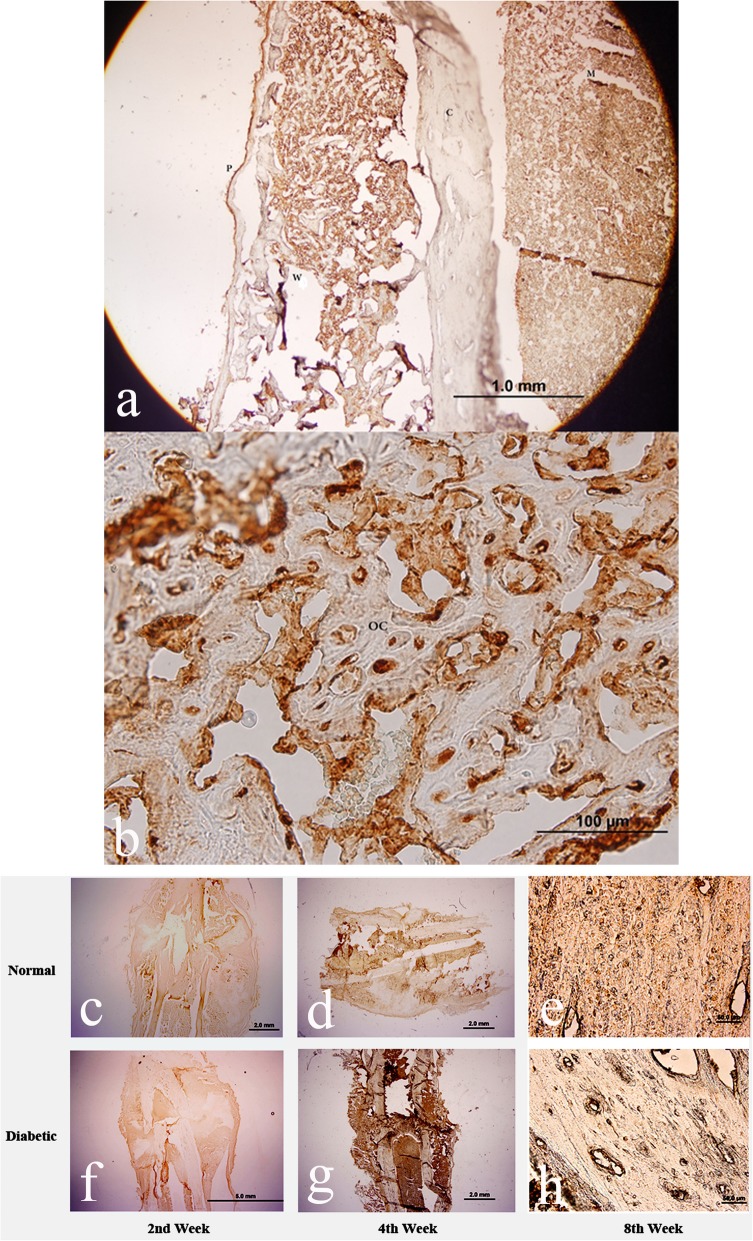
IHC of healing bone. BMP-6 in skeleton muscles (labeled as ‘m’), periosteum (as ‘p’), marrow and woven bone (as ‘w’) inside the callus. Positive spots were also found scattered in osteocytes (as ‘oc’) and extracellular matrix in cortex (as ‘c’). Fewer BMP-6 immunoreactive materials were detected in the cortex of the bone of the diabetic animals (**f**, **g**, **h**), compared with the non-diabetic ones (**c**, **d**, **e**)

The immunohistochemistry assay revealed that BMP-6 and BMP-9 were expressed in skeleton muscles, periosteum, marrow, and woven bone inside the callus. The immunoreactive positive spots were also found scattered in osteocytes and extracellular matrix in cortex (Fig. 4a, b showing expression of BMP-6). Fewer BMP-6 immunoreactive materials were detected in the cortex of the bones of the diabetic animals (Fig. 4f, g, and h), compared with the non-diabetic ones (Fig. 4c–e).

### Changes of BMP-6 and BMP-9

**Fig. 5:**
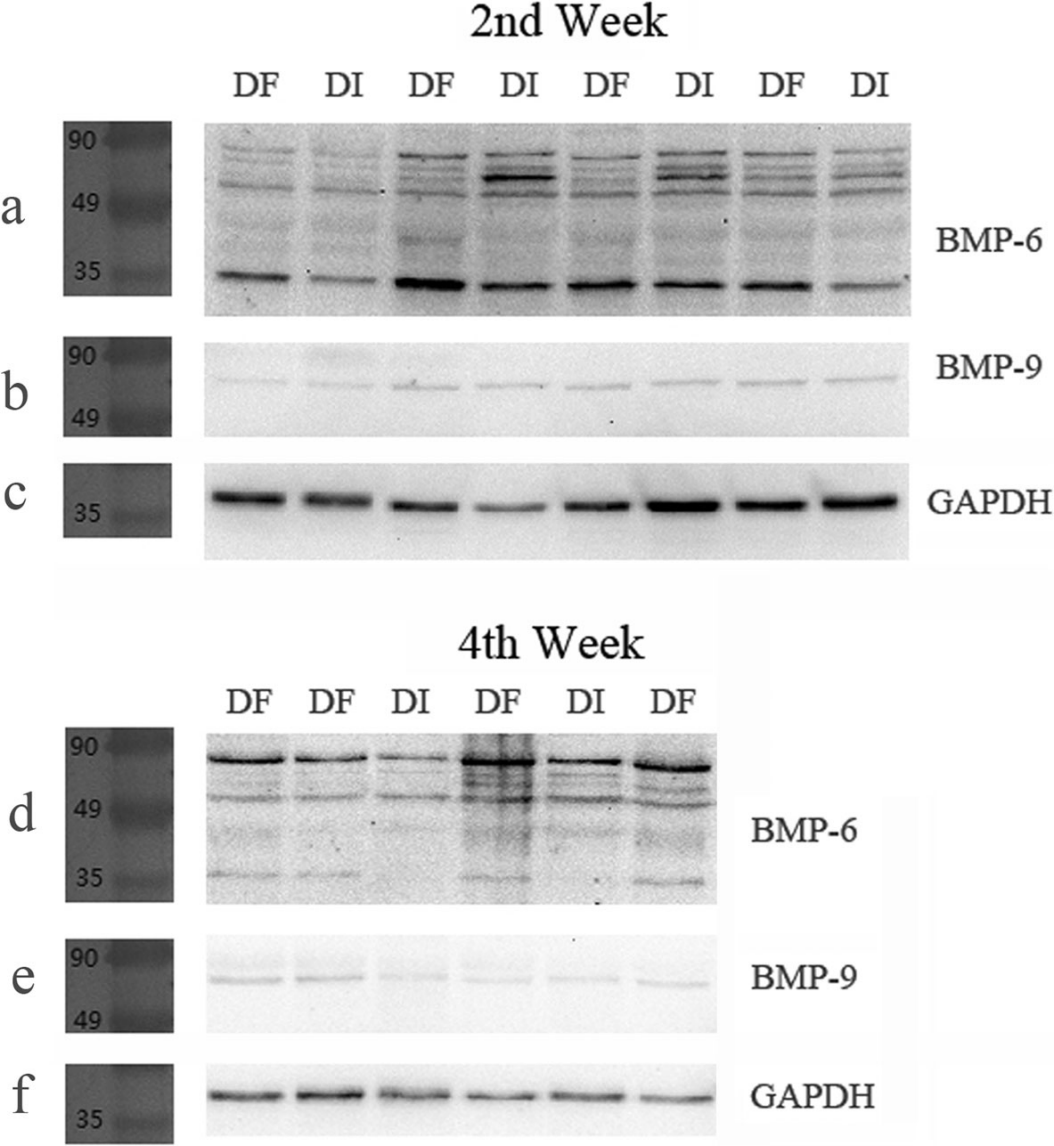
Western blot assay. Among the multiple bands of immunoreactive positive for BMP-6 (**a**, **d**), only the 35 kDa one was stably upregulated in fractured femurs at both the second and fourth post-fracture week; the only one band for BMP-9 was found, but not related to fracture (**b**, **e**)

**Fig. 6:**
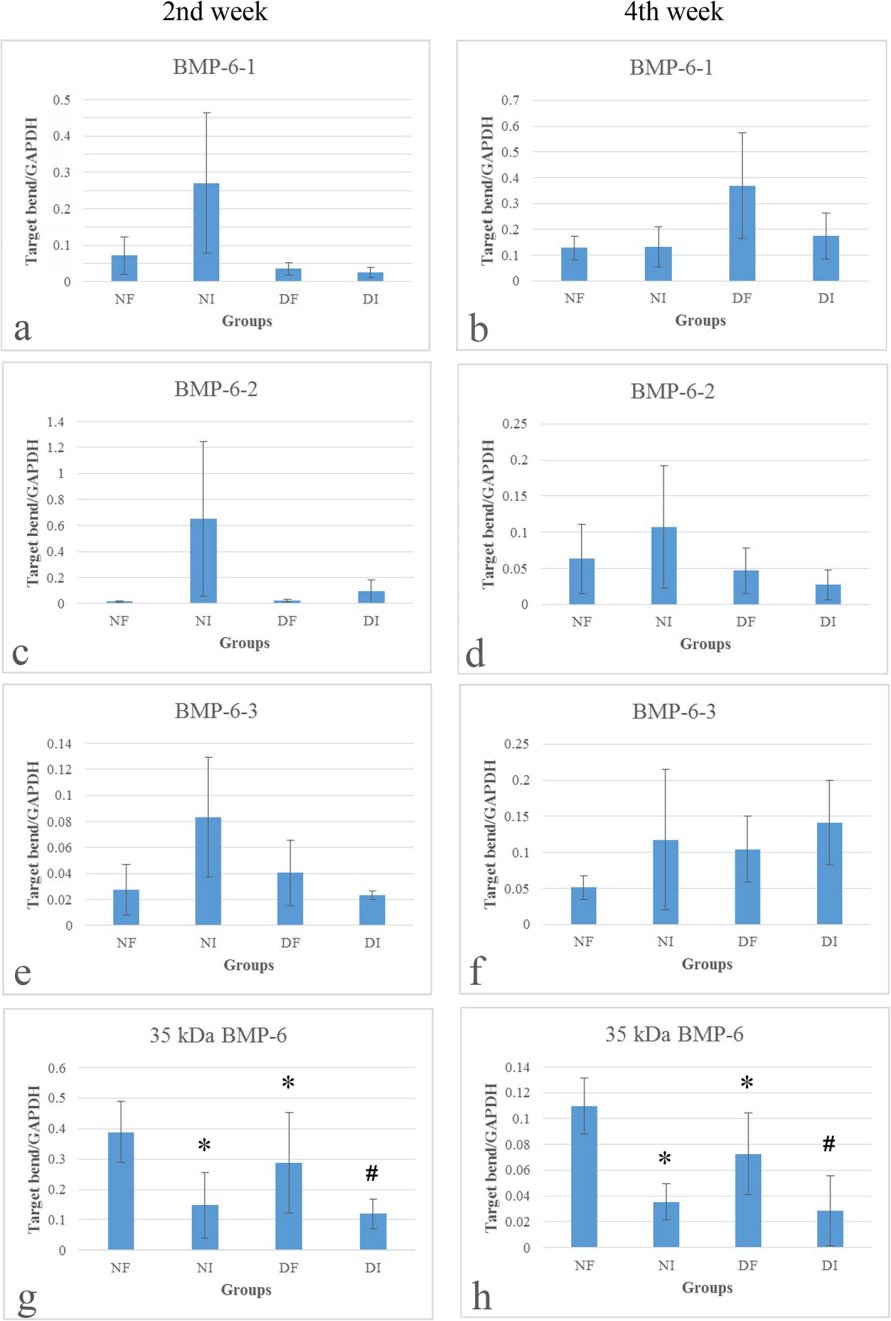
Quantification of 35 kDa BMP-6. 35 kDa BMP-6 was found upregulated in fractured femurs compared with intact femurs in both diabetic and non-diabetic animals (**g** and **h**, all *p* < 0.05). While in diabetic animals, 35 kDa BMP-6 was found significantly down-regulated, compared with non-diabetic ones in both fractured and intact femurs (all *p* < 0.05). *NF* normal fractured, *NI* normal intact, *DF* diabetic fractured, *DI* diabetic intact; *p < 0.05, compared with normal fractured; #*p* < 0.05, compared with diabetic fractured

**Fig. 7:**
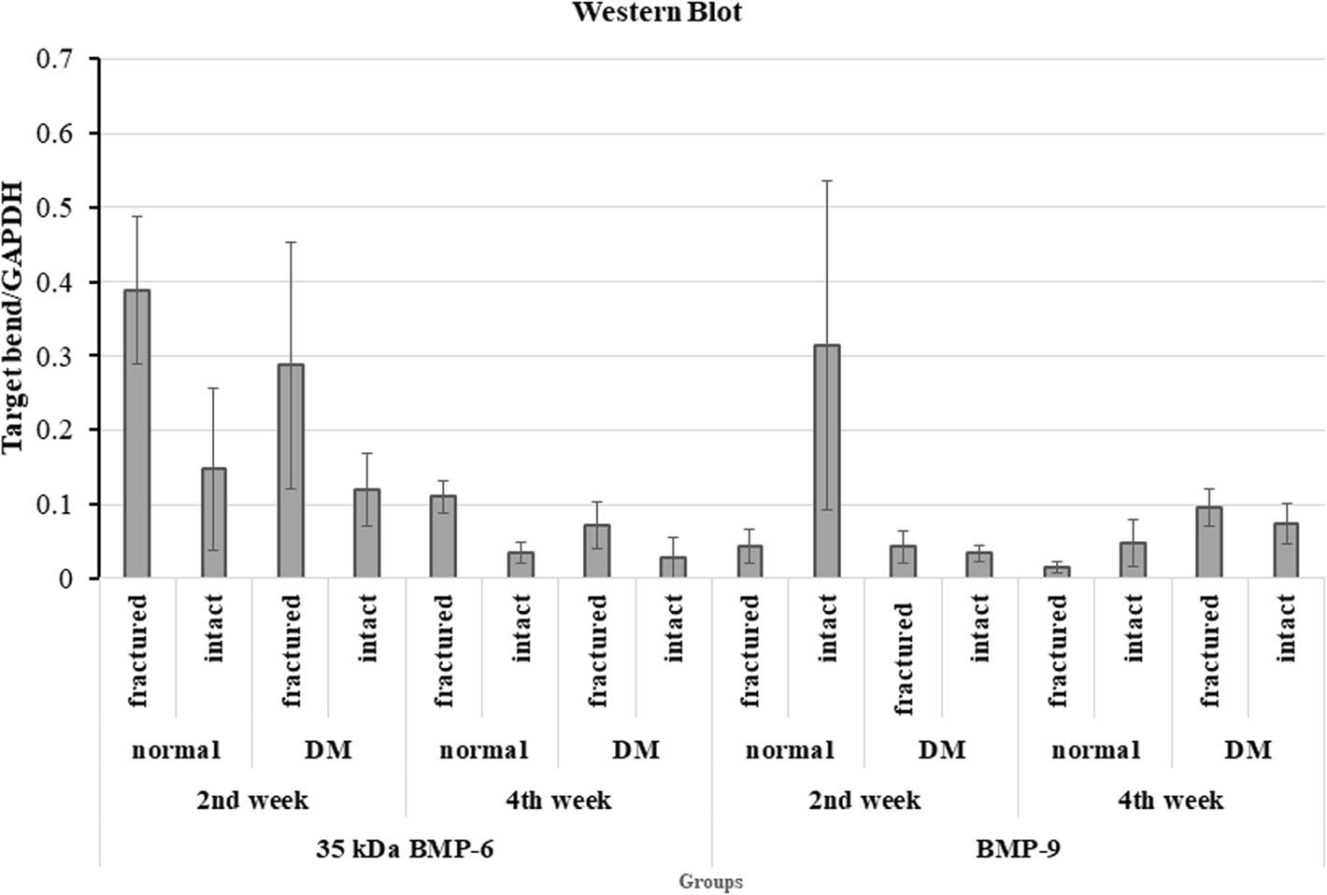
The impact of fracture and diabetes on BMP-6 and BMP-9. BMP-6 (35 kDa) was significantly upregulated in fractured femurs of both the normal and diabetic animals. Significantly lower level of BMP-6 (35 kDa) was observed in the fractured and intact femurs of the diabetic animals. No significant change in BMP-9 was detected among the fractured, intact, diabetic and non-diabetic animals

The results of Western blot assay showed multiple bands of immunoreactive positive to BMP-6. Four of them located between 49 and 90 kDa. One was between 35 and 49 kDa and another band was aligned with the marker of 35 kDa (Fig. 5a, d). The optical density values of all bands of different molecular weights were quantified and analyzed. Although the pattern of band layout was not strictly identical among each lane (sample), four most-frequently presented bands, including the three located between 90 and 45 kDa and the one with a molecular weight of 35 kDa, were compared and analyzed. As the exact molecular weight of the other three bands was unknown (not aligning with any of the molecular marker), here they were presented as BMP-6-1, -2, and -3 (in the order of molecular weights from high to low, as shown in Fig. 6a–f). Only the 35 kDa one was stably upregulated in fractured femurs at both the second and the fourth post-fracture weeks (Figs. 5a, c, d, f and 6g, h). However, only one band of immunoreactive to BMP-9 was found, but no relationship to fracture was detected (Figs. 5b, c, e, f and 7).

### Fracture and BMP-6

In comparison with the intact femurs, the expression of the BMP-6 (35 kDa) was significantly upregulated in fractured femurs of both the diabetic (Figs. 5a, c, d, f and 6g, h) and the non-diabetic rats (Fig. 6a–h). For the time-dependent expression differences, greater amount of expression of BMP-6 (35 kDa) was found at the end of the second week after fracture in both of the fractured and the non-fractured femurs of the diabetic (*n* = 5) and the non-diabetic animals (*n* = 5), when compared to those at the end of the fourth post-fracture week (diabetic group, *n* = 6; non-diabetic group, *n* = 6; Figs. 6g, h and 7, all *p* < 0.05). The findings indicate the participation of BMP-6 (35 kDa) in the healing of the fracture from the early time after fracture.

### Diabetes and BMP-6

However, the values of the BMP-6 (35 kDa) in both of the fractured and the non-fractured femurs from diabetic rats were much lower than their non-diabetic counterparts. Precisely, compared with the non-diabetic rats, the BMP-6 (35 kDa) in fractured and non-fractured femurs of the diabetic ones reduced by 25.95% (*n* = 5) and 19.15% (*n* = 5) respectively, at the end of the second post-fracture week, but by 34.01% (*n* = 6) and 18.83% (*n* = 6) respectively, at the end of the fourth week (all *p* < 0.05, Figs. 6g, h and 7). Therefore, it may indicate that the BMP-6 (35 kDa) participated in fracture healing, which was significantly inhibited by diabetes (by up to 34.01%).

### Ratio of BMP-6 molecules of different molecular weights

The ratio of the immunoreactive BMP-6 in forms of different molecular weights to the BMP-6 in 35 kDa was analyzed from the data collected from the animals of diabetic and non-diabetic group, at the end of the fourth week after femur fracture, to figure out the mechanism underlying the downregulation of the BMP-6 (35 kDa). The results showed significantly higher ratios in the BMP-6-1/BMP-6 (35 kDa) in diabetic ones (normal fracture vs. diabetic fracture, 1.19 ± 0.44 vs. 4.89 ± 1.69, *n* = 6, *p* < 0.01) and BMP-6-3/BMP-6 (35 kDa) (normal fracture vs. diabetic fracture, 0.47 ± 012 vs. 1.47 ± 0.43, *n* = 6, *p* < 0.01). The results indicate the impairment of the maturation of BMP-6 (35 kDa) in fractured bones of diabetic animals (Table 2).

**Table 2:** Ratio of optical density of the BMP-6 bands (M ± SD)

Groups	BMP6-1/35 kDa	BMP6-2/35 kDa	BMP6-3/35 kDa
Normal fracture	1.19 ± 0.44	0.55 ± 0.41	0.47 ± 0.12
Normal intact	3.85 ± 2.02&	2.77 ± 1.84&	3.50 ± 2.59&
Diabetic fracture	4.89 ± 1.69*	0.59 ± 0.25	1.47 ± 0.43*
Diabetic intact	5.17 ± 1.78	1.08 ± 0.51	4.61 ± 2.28#

^&^*p* < 0.01, compared with ‘Normal fracture’; *p < 0.01, compared with ‘normal fracture’; ^#^*p* < 0.05, compared with ‘Diabetic fracture’

## Discussions

It was not the first time that the connection between diabetes and fracture was studied. Skeleton and endocrine are not two isolated systems. Although the main function of bones is to support body weight, to provide attachments for muscles during movement, and to form cavities which protect organs, recent studies revealed that bone plays important roles in glucose and fat homeostasis and interacts with energy metabolism as an endocrine organ. For instance, osteocalcin, an osteoblast secreted protein, plays important roles in regulating bone metabolism and systemic glucose metabolism [18–21].

On the other hand, diabetes also has dramatic influence on bone formation and fracture healing. Terada and colleagues found that high glucose concentration impaired the proliferative response of osteoblasts to insulin-like growth factor-1 (IGF-1) and delayed osteoblast differentiation [22]. Sustained hyperglycemia, which is commonly seen in diabetic patients, increases formation of advanced glycation end product (AGE) which has been shown causing dose-dependent reduction of bone formation via the receptor of AGE [23]. The findings suggest that both hyperglycemia itself and its end products have negative effects on bone formation and fracture healing.

### Fracture healing and BMP-6

Although the potential participation of BMP-6 in bone formation was indicated in previous researches [18, 24–26], the role of the compound in diabetes-related impairment of fracture healing has not been elucidated. The novel finding of this study indicates that a particular member of the BMP-6 family, with a molecular weight of 35 kDa, may play an important role in fracture healing. The significantly higher (by 30–40 times) BMP-6 (35 kDa), detected at the end of the second post-fracture week, compared with that the fourth week, may indicate that the BMP-6 plays active roles in the early stage of fracture healing, i.e., chondrogenesis and early osteogenesis. Moreover, the early upregulation of the BMP-6 (35 kDa) may be derived by the post-translational mechanism, because neither difference in the transcription copies of the mRNA coding BMP-6 nor the BMP-6 proteins with higher molecular weights than 35 kDa was detected in previous studies [18, 24, 25].

### Diabetes and BMP-6

As the molecular weights of the other proteins showing immunoreactive positivity to the BMP-6 antibody were greater than the 35 kDa, if the BMP-6 at 35 kDa was the functioning form, the others (with greater molecular weights) could be the precursor and mid-products. Therefore, as the BMP-6 at 35 kDa was significantly upregulated in the fractured femurs, the volume of precursor and mid-products may be getting lower because of the shift of the precursor and the mid-products to the smaller functioning BMP-6 (35 kDa).

Our finding indicates that the transforming procedure from the molecules of greater molecular weights to the BMP-6 at 35 kDa was inhibited in diabetic animals. Therefore, the finding may suggests that diabetes downregulates the BMP-6 at 35 kDa through slowing down its maturing procedure. Obviously, the underlying mechanism is worth to be investigated.

Although the results in this study indicating BMP-6 (35 kDa) may play an important role in promotion of regeneration of bone and fracture healing, the findings reported here are primary, especially considering the limited sample sizes in this study, because of the ethic and economic consideration. Further investigation needs to be carried out targeting the mechanism underlying the pathogenesis of the delayed maturation of BMP-6 (35 kDa) and fracture healing, and the therapeutic approach via reversion of the pathological downregulation of BMP-6 (35 kDa).

In conclusion, downregulation of BMP-6 at 35 kDa may correlate with the impaired fracture healing in diabetes. The finding may suggests a potential target for the intervention of the pathological mechanism underlying the impairment of fracture healing in diabetes.

## Data Availability

The datasets used and/or analyzed during the current study are available from the corresponding author on reasonable request.

## Abbreviations

ANOVA: Analysis of variance
BMP: Bone morphogenetic protein
DF: Diabetic fractured
DI: Diabetic intact
DM: Diabetes mellitus
EDTA: Ethylene diamine tetraacetic acid
HE: Hematoxylin and eosin
IHC: Immunohistochemistry
kDa: Kilodaltons
NF: Normal fractured
NI: Normal intact
SD: Standard deviation
STZ: Streptozotocin
TGF-β: Transforming growth factor-β

## References

[1] International Diabetes Federation. IDF Diabetes Atlas, 9th edn. Brussels: 2019. Available at: https://www.diabetesatlas.org.

[2] Jiao H, Xiao E, Graves DT (2015). Diabetes and its effect on bone and fracture healing. Curr Osteoporos Rep.

[3] Vestergaard P (2007). Discrepancies in bone mineral density and fracture risk in patients with type 1 and type 2 diabetes-a meta-analysis. Osteoporos Int.

[4] Loder RT (1988). The influence of diabetes mellitus on the healing of closed fractures. Clin Orthop Relat Res.

[5] Hernandez PK, Do TP, Critchlow CW, Dent RE, Jick SS (2012). Patient-related risk factors for fracture-healing complications in the United Kingdom General Practice Research Database. Acta Orthop.

[6] Zura R, Xiong Z, Einhorn T, Watson JT, Ostrum RF, Prayson MJ (2016). Epidemiology of fracture nonunion in 18 human bones. JAMA Surg.

[7] Bishop JA, Palanca AA, Bellino MJ, Lowenberg DW (2012). Assessment of compromised fracture healing. J Am Acad Orthop Surg.

[8] Zura R, Mehta S, Rocca GJD, Steen RG (2016). Biological risk factors for nonunion of bone fracture. J Bone Joint Surg Rev.

[9] Botushanov NP, Orbetzova MM (2009). Bone mineral density and fracture risk in patients with type 1 and type 2 diabetes mellitus. Folia Med (Plovdiv).

[10] Stolzing A, Sellers D, Llewelyn O, Scutt A (2010). Diabetes induced changes in rat mesenchymal stem cells. Cells Tissues Organs.

[11] Sheweita SA, Khoshhal KI (2007). Calcium metabolism and oxidative stress in bone fractures: role of antioxidants. Curr Drug Metab.

[12] Gaston MS, Simpson AH (2007). Inhibition of fracture healing. J Bone Joint Surg Br.

[13] Ogasawara A, Nakajima A, Nakajima F, Goto K, Yamazaki M (2008). Molecular basis for affected cartilage formation and bone union in fracture healing of the streptozotocin-induced diabetic rat. Bone.

[14] Chen G, Deng C, Li YP (2012). TGF-β and BMP signaling in osteoblast differentiation and bone formation. Int J Biol Sci.

[15] Azad V, Breitbart E, Al-Zube L, Yeh S, O'Connor JP, S.S. Lin SS. (2009). rhBMP-2 enhances the bone healing response in a diabetic rat segmental defect model. J Orthop Trauma.

[16] Li H, Bian Y, Zhang N, Guo J, Wang C, Lau WB (2013). Intermedin protects against myocardial ischemia-reperfusion injury in diabetic rats. Cardiovasc Diabetol.

[17] Sun T, Guo Z, Liu CJ, Li MR, Li TP, Wang X (2016). Preservation of CGRP in myocardium attenuates development of cardiac dysfunction in diabetic rats. Int. J Cardiol.

[18] Fischerauer EE, Manninger M, Seles M, Janezic G, Pichler K, Ebner B (2013). BMP-6 and BMPR-1a are up-regulated in the growth plate of the fractured tibia. J Orthop Res.

[19] Hauschka PV, Lian JB, Cole DE, Gundberg CM (1989). Osteocalcin and matrix protein: vitamin K-dependent proteins in bone. Physiol Rev.

[20] Lee NK, Sowa H, Hinoi E, Ferron M, Ahn JD, Confavreux C (2007). Endocrine regulation of energy metabolism by the skeleton. Cell.

[21] Kanazawa I, Yamaguchi T, Yamamoto M, Yamauchi M, Kurioka S, Yano S (2009). Serum osteocalcin level is associated with glucose metabolism and atherosclerosis parameters in type 2 diabetes mellitus. J Clin Endocrinol Metab.

[22] Terada M, Inaba M, Yano Y, Hasuma T, Nishizawa Y, Morii H (1998). Growth-inhibitory effect of a high glucose concentration on osteoblast-like cells. Bone.

[23] Santana RB, Xu L, Chase HB, Amar S, Graves DT, Trackman PC (2003). A role for advanced glycation end products in diminished bone healing in type 1 diabetes. Diabetes.

[24] Kugimiya F, Kawaguchi H, Kamekura S, Chikuda H, Ohba S, Yano F (2005). Involvement of endogenous bone morphogenetic protein (BMP) 2 and BMP6 in bone formation. J Biol Chem.

[25] Yaoita H, Orimo H, Shirai Y, Shimada T (2000). Expression of bone morphogenetic proteins and rat distal-less homolog genes following rat femoral fracture. J Bone Miner Metab.

[26] Wang JF, Lee MS, Tsai TL, Leiferman EM, Trask DJ, Squire MW (2019). Bone morphogenetic protein-6 attenuates type 1 diabetes mellitus-associated bone loss. Stem Cells Transl Med.

